# Evaluating the Usability of an Emergency Department After Visit Summary: Staged Heuristic Evaluation

**DOI:** 10.2196/43729

**Published:** 2023-03-09

**Authors:** Hanna J Barton, Megan E Salwei, Rachel A Rutkowski, Kathryn Wust, Sheryl Krause, Peter LT Hoonakker, Paula vW Dail, Denise M Buckley, Alexis Eastman, Brad Ehlenfeldt, Brian W Patterson, Manish N Shah, Barbara J King, Nicole E Werner, Pascale Carayon

**Affiliations:** 1 Wisconsin Institute for Healthcare Systems Engineering University of Wisconsin-Madison Madison, WI United States; 2 Department of Industrial and Systems Engineering University of Wisconsin-Madison Madison, WI United States; 3 Center for Research and Innovation in Systems Safety Department of Anesthesiology Vanderbilt University Medical Center Nashville, TN United States; 4 Department of Biomedical Informatics Vanderbilt University Medical Center Nashville, TN United States; 5 School of Nursing University of Wisconsin-Madison Madison, WI United States; 6 University of Wisconsin-Madison Health Sciences Patient and Family Advisory Council Member Madison, WI United States; 7 Berbee Walsh Department of Emergency Medicine University of Wisconsin Hospital and Clinics Madison, WI United States; 8 Center for Aging Research and Education School of Nursing University of Wisconsin-Madison Madison, WI United States; 9 Department of Medicine School of Medicine and Public Health University of Wisconsin-Madison Madison, WI United States; 10 Berbee Walsh Department of Emergency Medicine School of Medicine and Public Health University of Wisconsin-Madison Madison, WI United States; 11 Department of Health and Wellness Design Indiana University School of Public Health-Bloomington Bloomington, IN United States

**Keywords:** patient safety, heuristic evaluation, usability, emergency medicine, safety, emergency, human factors engineering, usability, discharge summary, documentation, heuristic

## Abstract

**Background:**

Heuristic evaluations, while commonly used, may inadequately capture the severity of identified usability issues. In the domain of health care, usability issues can pose different levels of risk to patients. Incorporating diverse expertise (eg, clinical and patient) in the heuristic evaluation process can help assess and address potential negative impacts on patient safety that may otherwise go unnoticed. One document that should be highly usable for patients—with the potential to prevent adverse outcomes—is the after visit summary (AVS). The AVS is the document given to a patient upon discharge from the emergency department (ED), which contains instructions on how to manage symptoms, medications, and follow-up care.

**Objective:**

This study aims to assess a multistage method for integrating diverse expertise (ie, clinical, an older adult care partner, and health IT) with human factors engineering (HFE) expertise in the usability evaluation of the patient-facing ED AVS.

**Methods:**

We conducted a three-staged heuristic evaluation of an ED AVS using heuristics developed for use in evaluating patient-facing documentation. In stage 1, HFE experts reviewed the AVS to identify usability issues. In stage 2, 6 experts of varying expertise (ie, emergency medicine physicians, ED nurses, geriatricians, transitional care nurses, and an older adult care partner) rated each previously identified usability issue on its potential impact on patient comprehension and patient safety. Finally, in stage 3, an IT expert reviewed each usability issue to identify the likelihood of successfully addressing the issue.

**Results:**

In stage 1, we identified 60 usability issues that violated a total of 108 heuristics. In stage 2, 18 additional usability issues that violated 27 heuristics were identified by the study experts. Impact ratings ranged from all experts rating the issue as “no impact” to 5 out of 6 experts rating the issue as having a “large negative impact.” On average, the older adult care partner representative rated usability issues as being more significant more of the time. In stage 3, 31 usability issues were rated by an IT professional as “impossible to address,” 21 as “maybe,” and 24 as “can be addressed.”

**Conclusions:**

Integrating diverse expertise when evaluating usability is important when patient safety is at stake. The non-HFE experts, included in stage 2 of our evaluation, identified 23% (18/78) of all the usability issues and, depending on their expertise, rated those issues as having differing impacts on patient comprehension and safety. Our findings suggest that, to conduct a comprehensive heuristic evaluation, expertise from all the contexts in which the AVS is used must be considered. Combining those findings with ratings from an IT expert, usability issues can be strategically addressed through redesign. Thus, a 3-staged heuristic evaluation method offers a framework for integrating context-specific expertise efficiently, while providing practical insights to guide human-centered design.

## Introduction

### Overview

Heuristic evaluations are commonly used to evaluate the usability of health technologies [1,2]. Relying on human factors or usability experts to assess a technology against usability criteria (ie, heuristics), heuristic evaluations offer an efficient and low-cost alternative to user-based evaluation methods [3]. However, the method’s reliance on human factors expertise may limit its applicability and usefulness, especially regarding the evaluation of the severity of identified usability violations. In the domain of health care, usability violations can pose different levels of risk or harm to the patient; therefore, heuristic evaluation may require additional expertise besides human factors expertise [4,5]. One solution to this challenge is integrating other domains of expertise, such as clinical, patient and care partner, and IT expertise in the evaluation of a technology’s usability.

### Background

Selection of a list of criteria—whether referred to as guidelines, design principles, or heuristics—that constitute a “usable” technology is an essential aspect of conducting a heuristic evaluation. Molich and Nielsen’s [2] 1990 seminal article introducing heuristic evaluation included initial principles: simple and natural dialogue, speak the user’s language, minimize the user’s memory load, be consistent, provide feedback, clearly marked exits, shortcuts, good error messages, and error prevention. In practice, Nielsen’s [6] 10 heuristics, published online in 1995, are the most frequently used.

Typically, in conducting a heuristic evaluation, 1 expert reviews the technology looking for any and all violations of the selected usability criteria, producing a list of usability violations. Some identified violations are less significant than others, and as such, a follow-up step is often used to assess the severity of each violation to give direction for prioritization and redesign efforts. Upon initial conceptualization by Nielsen [7], a 5-step severity scale is often applied with scores that range from 0 (“not a usability problem at all”) to 4 (“usability catastrophe”).

### Adapting Heuristic Evaluation

Heuristic evaluations have been adapted for many domains and technologies, typically in one of the following ways: (1) the usability *criteria* on which the technology is evaluated, (2) the evaluation of the *severity* of usability violations, and (3) the *mode of conducting* the evaluation (eg, in groups) [3,5,8].

For example, Zhang et al [5] adapted the heuristic evaluation method for the assessment of medical devices, developing the Nielsen-Schneiderman heuristics. A synthesis of Nielsen’s 10 heuristics with Schneiderman’s “eight golden rules,” the 14 Nielsen-Schneiderman heuristics and their subbullets provide a comprehensive list of usability criteria that are especially useful in the evaluation of medical devices and health IT [5]. Another variation of the usability criteria is the ergonomic criteria defined by Scapin and Bastien [3]. They outline 8 categories of usability criteria: guidance, workload, explicit control, adaptability, error management, consistency, significance of codes, and compatibility. In contrast to the Nielsen-Schneiderman heuristics, the ergonomic criteria of Scapin and Bastien [3] provide a broader, macro-view of usability including consideration of workflow integration seen by their criteria “compatibility.”

Hermawati and Lawson [9] distinguish between general heuristics and heuristics developed for specific domains such as the evaluation of the usability of patient-facing documentation. For example, Tremoulet et al [8] conducted a heuristic evaluation of an emergency department (ED) after visit summary (AVS), the document handed to patients as they are discharged from the ED, that contains instructions and information to help them manage their symptoms, medications, and follow-up care [10]. Aiming to evaluate the usability of the AVS by outpatient clinicians (eg, clinicians supporting follow-up care), the authors adapted heuristic evaluation in a few ways. First, they selected usability criteria that integrated Nielsen’s heuristics with guidelines for effective health communication, so that the usability of the document could be more accurately assessed [8]. Further, consistent with participatory ergonomics principles, they partnered with clinical and human factors experts to conduct the evaluations [11]. In total, they identified 224 distinct usability issues across the 4 AVS documents they reviewed, of which 12 were considered catastrophic. For each of the AVS reviewed, content issues (eg, clarity of content, emphasis, context, relevance, and absence or lack of information) were the most common, accounting for 32% of the identified violations.

While Tremoulet et al [8] offer a domain-specific list of heuristics (ie, for the patient-facing AVS) and a compelling method for including clinical experts (eg, primary care physicians) in the heuristic evaluation of patient-facing documents, there remains a gap in understanding the usability of the AVS from the patient’s perspective. This is important as the patient is the one who will ultimately receive the AVS (presumably), carry it home, and (possibly) interact with it after discharge from the ED. Further, the AVS has been identified as an important tool for care coordination between the ED and the home—a transition that is highly vulnerable to patient safety problems [10,12,13].

Therefore, in evaluating the usability of patient-facing documents, it is important to include the perspective of patients and care partners, as well as the perspective of clinicians who interact with patients and care partners in sharing and reviewing those documents. In addition, the heuristic evaluation can produce more impactful results if the violations are evaluated for potential redesign; this calls for the involvement of IT experts who can provide important information on whether violations can be addressed in the redesign phase. Thus, adapting heuristic evaluation methods to efficiently incorporate expertise from clinicians, patients and care partners, and IT professionals is necessary to assess and address potential impacts on patient safety.

### Research Objective

The objective of this study was to assess a method for integrating diverse expertise (ie, clinical, patient and care partner, and IT) with human factors engineering (HFE) expertise in the evaluation of an ED AVS.

## Methods

### Overview

This study was part of an AHRQ Patient Safety Learning Lab aimed at developing a set of tools to improve care coordination for older adults who come to the ED with a fall or suspected urinary tract infection [14]. As part of the development of an intervention to improve the discharge process for patients transitioning to the home, we recognized the need for an initial assessment of the patient-facing ED AVS. As such, we conducted a 3-staged heuristic evaluation (Table 1) of 2 versions of an ED AVS to inform the design and implementation of a patient-centered discharge process. This work was done early in the COVID-19 pandemic, and therefore, was conducted digitally via videoconferencing software.

**Table 1:** Three-staged heuristic evaluation method.

Stage	Expertise	Guiding questions	Process
1	HFE^a^	What usability issues exist in the current AVS^b^? What heuristics do they violate?	1.5-hour meeting with 8 HFE experts facilitated by 1 researcher (HJB) Sent preparation materials to HFE experts: key literature, the AVS documents being evaluated, list of heuristics, and an example output of a heuristic evaluation Using the AVS documents provided and moving from left to right and from top to bottom, the group identified usability issues and the specific heuristics they violated
2	Clinical (emergency medicine, geriatrics, and nursing) patient and care partner	What issues have the largest impact on patient safety and comprehension? What do we need to address first?	Six participants: 2 emergency medicine physicians, 1 ED^c^ nurse, 1 nurse with transitional care expertise, 1 geriatrician, and 1 older adult care partner Participants rated each usability issue as having “no impact,” “some negative impact,” or “large negative impact” on our 2 criteria: patient comprehension and patient safety (~1 hour) 30-minute one-on-one debrief with each participant led by HFE team members (HJB and PC) to resolve outstanding questions and capture additional usability issues
3	Health IT	What issues can we address?	IT expert scored each violation as “can be addressed,” “maybe,” or “impossible to address” in response to the prompt: “How likely are we (from an IT perspective) to be able to address this violation?” (~1 hour) 30-minute one-on-one debrief with participants led by HFE team members (HJB and PC) to ask clarifying questions

^a^HFE: human factors engineering.

^b^AVS: after visit summary.

^c^ED: emergency department.

### Selection of Heuristics

Heuristics for evaluating the ED AVS were selected by comparing Tremoulet et al’s [8] domain-specific heuristics to 2 prominent sets of heuristics, discussed in the introduction: (1) Scapin and Bastien’s [3] list of ergonomic criteria and (2) the Nielsen-Schneiderman heuristics developed by Zhang et al [5]. The results of this comparison demonstrated that the Tremoulet et al [8] heuristics were comprehensive, and yet tailored for the evaluation of paper-based, patient-facing documentation. Thus, we selected the Tremoulet et al [8] heuristics, modifying them slightly to include questions from the associated Scapin and Bastien [3] and Nielsen-Schneiderman heuristics (Table 2).

**Table 2:** List of heuristics used in this study based on Tremoulet et al [8] heuristics augmented by Scapin and Bastien [3] and Nielsen-Schneiderman heuristics [5] (denoted in italics).

Heuristic categories and names		Description
**Readability: The information is presented in a manner that is easy to read.**		
	Color and contrast	Does the text have sufficient contrast?
	Layout and position	Is the layout appealing, clear, and consistent across the document?
	Font and capitalization	Are the font and its size consistent and readable?
	Structure and format	Are the structure and format of each section effective and uniform?
**Minimalism: Information is presented as simply and succinctly as possible.**		
	Simple and direct	Are the language and sentence structure simple, direct, specific, concrete, and concise? *Note: Simple is not equivalent to abstract and general*
	Progressive level of detail	Does the document present the most important information first, following with increasing levels of detail?
**Comprehensibility: It is easy for the reader to make sense of the information that is presented.**		
	Terminology	Are complex and technical terms used correctly and consistently? *Are standard meanings of words used? Is language from the users’ perspective?*
	Clarity of headings	Are the headings clear and understandable?
**Content: All the information that is presented is relevant to either a clinical expert or the** **older adult care partner,** **and no information needed by either of these parties is missing.**		
	Clarity of content	Is the purpose of the material obvious?
	Emphasis	Are important points emphasized appropriately? Is it clear why certain text is emphasized?
	Context	Does the document include the creation or printing date and contact information?
	Relevance	Is the content relevant to the patient’s condition and context? Is there extraneous information?
	Absence or lack of information	Is any important content missing?
**Organization: Information is ordered logically and grouped into reasonably sized sections with prominent and meaningful headings and subheadings.**		
	Grouping	Is the information grouped in a meaningful format? Are the groups reasonably sized? *Is there clear visual distinction between sections?*
	Order	Is the information ordered logically? *Is like content grouped together?*
	Use of subheadings	Does the document use prominent and meaningful headings and subheadings?
	Navigational tools	Does the material have navigational tools to help orient the reader? *Is context-sensitive help embedded in the contents?*

### Selection of ED AVS

For our heuristic evaluation, an ED AVS was simulated with fake patient data. In addition, we evaluated a redacted real-life ED AVS provided by the care partner who participated in our study. Using the second ED AVS allowed us to identify any usability issues that were artifacts of the simulation.

### Stage 1: HFE Experts Identify Usability Issues

To identify usability issues, a group of 8 HFE experts met for 1.5 hours on June 23, 2020, to review the 2 AVS. Before conducting the evaluation, all participants were asked to review Tremoulet et al [8] article, the finalized list of heuristics (Table 2), the AVS documents being evaluated, and an example of a final report produced from a past heuristic evaluation. Additional heuristics literature was provided for the participants to review if they elected to [2,5].

During the virtual meeting, 1 researcher served as a facilitator (HJB)—sharing their screen and guiding the group through the ED AVS document from top-to-bottom and left-to-right. All participants were encouraged to verbalize the usability issues they viewed. Once an issue was identified, participants worked collaboratively to name the associated heuristics violated. When issues were identified, the facilitator circled them on the shared view of the ED AVS and numbered them for ease of reference.

The final list of identified usability issues and their associated heuristic violations was reconciled by researchers (HB, KW, and RR) within 24 hours of the group meeting. Snipped images of the marked-up ED discharge summaries were taken to give context for each of the issues identified.

### Stage 2: Clinical, Patient, and Care Partner Experts Rate the Impact of Usability Issues

We selected a variety of experts to assess the impact of the identified usability issues on patient comprehension and safety. These experts include emergency medicine physicians (n=2), an ED nurse (n=1), a nurse with transitional care expertise (n=1), a primary care geriatrician (n=1), and an older adult care partner (n=1).

The type of expertise each expert provided was unique. The care partner referred to their perspective as an older adult and their lived experience having previously visited the ED with their partner 14 times over the course of 10 weeks. The emergency medicine physicians and ED nurse used their clinical expertise; the ED nurse also referred to nurses’ experiences reviewing the AVS with patients and their care partners as they are being discharged from the ED. Further, a nurse with expertise in older adult transitions and a geriatrician provided perspective on how patients and their care partners interact (or do not interact) with the AVS after discharge from the ED, including in the context of an outpatient follow-up visit.

Each expert was asked to rate each identified usability issue’s impact on 2 criteria using a 3-point scale (ie, no impact, some negative impact, or large negative impact). The 2 criteria, selected through discussion and review of the literature, were (1) patient comprehension and (2) patient safety [15,16]. We defined patient comprehension as “the patient’s understanding of the information, for example, what to do next, what to watch for, and what to expect” and patient safety as “the patient’s ability to follow-up and follow-through with recommendations.” As such, patient safety would be negatively impacted by any usability issue that could result in a lack or delay of follow-up, taking the wrong actions, or potential patient harm.

In addition to providing ratings on each criterion for each usability issue, we asked experts to take note of any usability issues that were unclear to them and identify any additional usability issues they may have noticed in the AVS documents that were not identified in stage 1. Each expert’s ratings and notes were then sent back to the research team. One researcher (HJB) reviewed each expert’s ratings and notes for missing data, newly identified usability issues, and any notes of interest. A 30-minute final debrief meeting was scheduled with each expert, wherein researchers (HJB and PC) met with each expert to collect any missing data, ask clarifying questions, and capture any other feedback on the process. Five experts’ ratings and interviews were conducted in August 2020. The final expert’s rating and interview, the geriatrician’s, were conducted in October 2020.

Impact ratings were then converted to a numerical score (0=no impact; 1=some negative impact; 2=large negative impact) for comparison and analysis. Average scores on each criterion were calculated for every usability issue.

### Stage 3: IT Expert Assesses the Likelihood of Addressing Usability Issues

In the third stage, an electronic health record (EHR) architect from our partner health care organization with extensive institutional knowledge rated each usability issue on the “likelihood we would be able to address it” using a 3-point scale (ie, impossible to address, maybe, or can be addressed). In addition, the IT expert was asked to take note of any comments related to their responses. The expert’s ratings and comments were reviewed by a researcher (HJB) prior to a 30-minute final debrief meeting with researchers (HJB and PC) to discuss ratings and associated comments with the IT expert. Stage 3 was completed in September 2020.

### Ethical Considerations

This study procedure was exempt from IRB approval as part of a quality improvement initiative. There was no compensation for participation.

## Results

### Usability Issues and Their Associated Heuristic Violations

In stage 1, we identified 60 unique usability issues, violating a total of 108 heuristics (each usability issue could violate more than 1 heuristic). We identified violations for each of the categories of heuristics except for 2 heuristics: readability—color and contrast and content—context. The number of violations per heuristic ranged from 0 to 16 (Table 3), with the most frequently violated being clarity of content (16 of 108), absence or lack of information (15 of 108), relevance (13 of 108), and grouping (11 of 108).

In stage 2, clinical, patient, and care partner experts identified 18 additional usability issues, violating an additional 27 heuristics, including the 2 categories of heuristics not identified in stage 1. The number of violations per heuristic ranged from 0 to 7, with 5 heuristics with no new violations identified by our experts (Table 3).

In total, we identified 78 unique usability issues, violating a grand total of 135 heuristics. The heuristics most frequently violated were absence or lack of information (n=22), clarity of content (n=19), relevance (n=14), and terminology (n=12). All heuristics were violated at least once.

**Table 3:** Number of heuristic violations identified by stages.

Heuristic categories and names		Heuristic violations identified in stage 1 (n=108), n	Heuristic violations identified in stage 2 (n=27), n	Total heuristic violations identified (N=135), n
**Readability**				
	Color and contrast	0	1	1
	Layout and position	4	0	4
	Font and capitalization	5	2	7
	Structure and format	2	1	3
**Minimalism**				
	Simple and direct	4	4	8
	Progressive level of detail	3	1	4
**Comprehensibility**				
	Terminology	10	2	12
	Clarity of headings	5	0	5
**Content**				
	Clarity of content	16	3	19
	Emphasis	5	1	6
	Context	0	3	3
	Relevance	13	1	14
	Absence or lack of information	15	7	22
**Organization**				
	Grouping	11	0	11
	Order	9	0	9
	Use of subheadings	5	1	6
	Navigational tools	1	0	1

### Impact Ratings of Usability Issues

In stage 2, we sought to determine the impact of each usability issue on two criteria: (1) patient comprehension and (2) patient safety. We found that average scores on both criteria ranged from 0 (eg, all experts rated “no impact”) to 1.83 (eg, 5 out of 6 experts rated “large negative impact”). The highest rated usability issues included, for example, that “there [was] no indication as to whether the medication list [was] up-to-date, or even if it was reviewed by the ED” (Table 4). This issue scored 1.5 on the patient comprehension criterion and 1.67 on the patient safety criterion. Additional examples are included in Table 4.

Further, we wanted to see if there were differences between the experts’ impact ratings. It was found that on average the older adult care partner used the rating “large negative impact” more frequently than the clinical experts—for example, 37 times when rating usability issues on patient comprehension; the next most used being 23 times (Table 5). Finally, a significant correlation between our 2 criteria, patient comprehension and patient safety, were identified but not between any participant ratings (eg, there was no significant correlation between the 2 ED physicians on either criterion).

**Table 4:** Highest rated usability issues, the heuristics they violate, their average impact scores on patient comprehension and patient safety, and their likelihood of being addressed.

	Highest-rated usability issues	Heuristics violated	Average impact score on patient comprehension	Average impact score on patient safety	Likelihood of being addressed
1	The section “what’s next” is similar to the “instructions” section and presents conflicting information from what is listed under “instructions.” It is unclear to what extent the “what's next” section relates to the “follow-up” section.	Terminology Simple and direct Grouping Clarity of headings	1.83	1.83	Impossible to address
2	The first page of the AVS^a^ document is cluttered and the information is not presented in a way that makes sense.	Use of subheadings Progressive level of detail Grouping	1.83	1.5	Impossible to address
3	AVS is written at a high comprehension level. No visuals or graphics to support comprehensibility. No contact for services that could support people with low reading comprehension (eg, cognitive impairments and nonnative English speakers)	Absence or lack of information Simple and direct	1.67	1.67	Impossible to address
4	No instructions to follow-up to have wound checked or stitches removed (or who to do this with). The only follow-up mentioned is with rehab and they are not going to do this.	Absence or lack of information Context	1.67	1.67	Can be addressed
5	The “what's next” section needs to include a list of the tasks that the patient needs to do next. It should also be grouped with “follow up.”	Absence or lack of information Grouping	1.5	1.67	Impossible to address
6	There is no indication as to whether this medication list is up-to-date, or even if it was reviewed by the ED.^b^	Context Absence or lack of information	1.5	1.67	Maybe

^a^AVS: after visit summary.

^b^ED: emergency department.

**Table 5:** Average impact scores and the number of highly rated usability issues by experts.

			Older adult care partner representative		Nurse with transitional care expertise	Geriatrician^a^		Emergency medicine physician 1		Emergency medicine physician 2		ED^b^ nurse		Average
**Patient comprehension (n=76 usability issues)**														
	Average impact score	1.197		1.080		1.026	1.184		0.882		0.789		1.026	
	Usability issues rated “large negative impact” (eg, score=2), n	37		17		23	23		6		9		19	
**Patient safety (n=76 usability issues)**														
	Average impact score	1.276		1.120		0.961	0.816		0.421		0.645		0.872	
	Usability issues rated “large negative impact” (eg, score=2), n	42		32		26	14		3		9		21	

^a^The geriatrician rated 78 usability issues. All other experts rated 76 usability issues.

^b^ED: emergency department.

### Likelihood of Addressing Usability Issues

In stage 3, an IT expert from our partner health system with extensive experience with the ED AVS provided ratings on the “likelihood we would be able to address” each usability issue. Of the 76 usability issues that the expert reviewed, 31 usability issues were rated as “impossible to address,” 21 as “maybe,” and 24 as “can be addressed.” The reasons most cited for being unable to address a usability issue were because the information in the AVS came from an outside vendor (eg, generic patient instructions for wound care) or because the EHR vendor controlled the headers, content, and order of the sections. The reasons cited for why a usability issue may be able to be addressed were because a solution would require additional work for clinicians (eg, ED physicians and nurses) or because it would require an overhaul of the databases that populate the AVS (eg, the name of the clinic to follow-up with). Finally, the usability issues that were most often cited as being able to be addressed were the ones found in sections that the health organization had added to the AVS (eg, generic reminders to wear a seatbelt).

## Discussion

### Overview

This study found that it is important to integrate diverse expertise to evaluate usability when patient safety is at stake. Twenty-three percent of the identified usability issues (18/78)—a large proportion of which were related to the absence or lack of information—were noted by clinical, patient, and care partner experts in stage 2 and would not have otherwise been identified by HFE experts. The additional 18 usability issues identified by non-HFE experts represent the need to integrate a broader range of expertise.

To conduct a comprehensive heuristic evaluation, expertise from all *contexts* of use must be considered. In the case of the ED AVS, the experts included (1) the emergency medicine physician who initiates the creation of the AVS in the EHR, but rarely ever sees it printed out; (2) the ED nurse who prints out the AVS and reviews and discusses it with the patient and their care partner upon discharge from the ED; (3) the patient and care partner who receive the document from the ED nurse, carry it home, and who may need to communicate about it with other care partners, family, and their doctor; and (4) the geriatrician (or other primary care doctor) who hears about the ED visit from the patient during their follow-up and may or may not interact directly with the AVS. Thus, the usability of the AVS may differ between the multiple distinct contexts of use. Methods that capture the complex and, on occasion, conflicting perspectives of relevant experts are required to appropriately assess usability and inform redesign.

Similar to findings from a study comparing clinician and patient ratings of nonroutine events, our results demonstrate discrepancies in the impact ratings of different experts [17]. Particularly, the older adult care partner rated usability issues as having a more negative impact on patient comprehension and patient safety. The scores from the geriatrician and nurse with transitional care expertise were similarly high, which may point to poorer usability of the AVS in post ED discharge contexts [18]. Including these *context-specific* experts in evaluating the impact of the identified usability issues aligns the design priorities with the experience of patients and their care partners upon leaving the ED. Aligning design priorities with the experience of patients and their care partners is a key aspect of designing patient-centered systems [19].

These initial steps at capturing a variety of context-specific expertise point to a unique challenge: How do we integrate these perspectives and choose where to focus our design efforts? This reconciliation of multiple perspectives is a pervasive challenge for diverse health care design teams [20]. One way to address this is by clearly defining an aim, for example, design a patient-centered discharge process, that can guide the integration and prioritization of perspectives in a design team with representation from multiple stakeholders. HFE methods such as participatory design and co-design offer frameworks for doing this [21-24].

The 3-staged method introduced in this paper also begins to bridge the gap between heuristic evaluation and redesign. Capturing insight from an IT expert in stage 3 about what it would take to address each identified usability issue provides practical feedback that can be incorporated into a redesign process. Further, an EHR architect, in particular, may provide insight into the level at which each usability issue could be addressed, for example, at the health system level or at the level of the EHR vendor. By engaging IT during the evaluation of the AVS versus later in the design process, resources can be used more efficiently. Furthermore, given the challenges, frontline staff must upskill well-designed, usable technologies; this method may also bridge the gap from redesign to implementation by avoiding designing a solution that cannot be implemented [25].

### Lessons Learned

Our staged method for heuristic evaluation produced uniquely practical insight while remaining efficient. The staged approach allowed for the combined benefit of group heuristic evaluation, that is, the inclusion of multiple HFE experts during initial usability issue identification and the efficient solicitation of feedback from stakeholders with their unique expertise.

#### Time Investments

The 7 non-HFE experts whose feedback was obtained in stages 2 and 3 spent between 1.5 and 2 hours in total reviewing the usability issues on their own and then debriefing with 2 HFE experts. Four of the HFE experts contributed solely to the stage 1 meeting, that is, 1.5 hours of their time; 2 additional HFE experts contributed an additional 2-3 hours of support in taking notes and preparing an initial report of usability issues after the stage 1 meeting. The remaining 2 HFE experts were heavily involved in the preparation for and execution of all 3 stages, for example, communicating and scheduling with experts, reviewing expert’s feedback, debriefing, and so forth.

#### Role of HFE Experts

Given our staged approach, HFE experts played different roles at different points in time. During stage 1, HFE experts were the main source of identifying usability issues and assessing which heuristics those issues violated. During stages 2 and 3, HFE experts served more as facilitators to capture insights from other non-HFE experts and translate them into usability issues, heuristic violations, and relevant feedback on our ability to address those issues.

#### Selection of Experts

An important aspect of this study is the selection of experts who have relevant context-specific expertise. For example, to represent the interest of a primary care doctor who would follow-up with a patient post ED visit, we selected a geriatrician who is likely to see patients from the population we are designing for, that is, older adults (65+ years) with a recent fall or urinary tract infection. Further, in selecting the IT expert for stage 3, their extensive experience with the ED AVS, as in, how it has been changed over time by the EHR vendor and by the health system, and the processes through which it gets changed within the health system, was essential to providing useful data.

### Limitations

A few limitations of this study should be noted. First, given this study was not designed to be generalizable, we used small sample sizes, for example, 6 experts that provided feedback during stage 2. Future work could more extensively explore the discrepancies between experts’ perspectives by increasing the sample size. These data may alter how relevant one considers a single type of expert’s perspective to be, for example, if there is little significant difference between certain experts. Particularly, additional patient and care partner perspectives may be warranted to capture the variety of experiences patients have based on their identity, cognitive abilities, living situation, and so forth.

### Conclusions

Capturing relevant context-specific expertise in heuristic evaluation results in more comprehensive identification of usability issues and their impacts. Despite being challenging to integrate, experts’ unique perspectives must be considered to design patient-centered systems. A staged approach to heuristic evaluation may be a useful tool to more reliably identify usability issues that are significant in the patient experience and translate those into actionable redesign.

## Abbreviations

AVS: after visit summary
ED: emergency department
EHR: electronic health record
HFE: human factors engineering

## Data Availability

Data from this study are not publicly available.
